# Fibromyalgia: Neuropsychological and Clinical Correlates in Suicidal Behavior Based on Ideation-to-Action Models—A Critical Review

**DOI:** 10.3390/bs16020258

**Published:** 2026-02-10

**Authors:** Cristina Muñoz Ladrón de Guevara, Sandra Melero

**Affiliations:** Department of Psychology, Faculty of Education Sciences, University of Cádiz, 11519 Puerto Real, Spain

**Keywords:** fibromyalgia, suicidal behavior, neuropsychological alterations, clinical correlates, ideation-to-action models

## Abstract

Fibromyalgia (FM) is associated with increased suicidal behavior (SB). This critical review integrates the ideation-to-action models—Interpersonal Theory of Suicide (IPTS), Three-Step Theory (3ST), and Integrated Motivational–Volitional (IMV) Model—with clinical and neuropsychological correlates to discriminate between suicidal ideation (the motivational component) and suicidal action (the volitional component) in FM. Ideation is related to hopelessness, perceived burden, thwarted belongingness, and entrapment, as well as to pain/interference, sleep disturbances, fatigue, mood, pain catastrophizing, and attentional pain vigilance. The transition to action is associated with impulsivity, executive dysfunction (including inhibitory control, flexibility, and decision-making under ambiguity/risk), acquired capability due to repeated exposure to pain and medical procedures, and access to lethal means. Suicidal planning is conceptualized as high-severity ideation, while action includes preparatory behaviors and suicide attempts. Evidence from Spanish instruments is synthesized—Columbia Suicide Severity Rating Scale (C-SSRS), Plutchik Suicide Risk Scale (PSRS), Beck Depression Inventory-II (Item 9 of the BDI-II), and Suicide Behaviors Questionnaire—Revised (SBQ-R)—pointing out overlaps with pain/depression and the lack of specific validation in FM. Prospective cohorts, standardization of definitions/windows, comparable neuropsychological batteries, and mechanistic trials on motivational and volitional targets and interventions focused on pain reduction are proposed.

## 1. Introduction

Fibromyalgia syndrome or fibromyalgia (FM) is defined as a chronic, generalized, and diffuse musculoskeletal pain disorder, widely conceptualized as a prototypical nociplastic pain condition (International Association for the Study of Pain, n.d.), not associated with obvious clinical inflammation, tissue damage, or deformity (Chinn et al., 2016; Wolfe et al., 1995, 2010). Although its etiology and pathophysiology remain incompletely understood (Benjamin et al., 2025; Peck et al., 2020), converging evidence supports a robust mechanistic framework of central sensitization and altered pain processing, including dysfunctions in descending pain modulation (Bhargava & Goldin, 2025; Clauw, 2009, 2015; Woolf, 2011; Carneiro et al., 2025b). Despite the absence of clinical or systemic signs of inflammation and structural damage, and the controversy this generates, various investigations have described signals compatible with low-grade inflammation and possible neuroinflammation, characterized by elevated pro-inflammatory cytokines and immune dysregulation. However, the evidence is heterogeneous and inconclusive; these processes could contribute to both central and peripheral sensitization (Baral et al., 2019; García-Domínguez, 2025; Mueller et al., 2023; Siracusa et al., 2021).

Regarding the epidemiology, studies indicate a higher prevalence in women than in men, with onset most frequently between 30 and 35 years of age, and general population rates ranging from 2.0% to 5.0% (with variability based on criteria and country) (Jurado-Priego et al., 2024; Siracusa et al., 2021; Wolfe et al., 1995, 2010). Furthermore, FM is the third most common musculoskeletal disorder, after low back pain and osteoarthritis (Sarzi-Puttini et al., 2020). Clinically, it is characterized by allodynia, hyperalgesia, morning stiffness, persistent and debilitating fatigue, anxiety, depression, non-restorative sleep, and cognitive deficits (e.g., “fibro-fog”) (Wolfe et al., 1995, 2010). This symptomatic pattern severely limits occupational, social, and family functioning, and leads to a high consumption of healthcare resources. Compared to other rheumatological diseases, FM has a particularly negative impact on the emotional, social, intellectual, and health domains, dramatically reducing the quality of life of those affected (Cabo-Meseguer et al., 2017; Gálvez-Sánchez et al., 2024). Moreover, it is associated with an increased risk of suicidal behavior (SB) (Adawi et al., 2021).

**Aims and Scope:** This critical review analyzes the relationship between FM and SB, with the latter composed of ideation plus suicidal action (see below). It is limited to the adult FM population and emphasizes neuropsychological variables (executive functions, impulsivity, decision-making under risk/ambiguity) alongside pain-related cognitive/attentional processes (pain catastrophizing, attentional pain vigilance) and their interaction with pain interference, sleep disturbances, and fatigue, among others. The synthesis is organized using ideation-to-action models (Interpersonal Theory of Suicide [IPTS], Three-Step Theory [3ST], and Integrated Motivational–Volitional Model [IMV]) to delineate their contribution to the genesis of ideation (hopelessness, perceived burden, thwarted belongingness) and the transition from ideation to attempt (executive control dysfunction, impulsivity, acquired capability).

In contrast to previous systematic reviews and meta-analyses on suicidal behavior in FM—which have primarily focused on prevalence/risk estimates and broad clinical or psychopathological correlates (e.g., Levine & Horesh, 2020; Adawi et al., 2021; Treister-Goltzman & Peleg, 2023)—this review makes a distinct contribution by synthesizing mechanistic evidence through an explicit ideation-to-action lens. Rather than updating pooled prevalence estimates, we map the literature onto ideation-to-action processes to clarify how candidate mechanisms may differentially relate to the emergence of suicidal ideation versus progression to suicidal action in FM.

This work adopts a non-systematic critical review format, integrating clinical evidence, neuropsychological data, and theoretical models to derive practical implications and research priorities. For the first time explicitly to the best of our knowledge, this review articulates the ideation-to-action models with neuropsychological correlates and clinical variables in FM to explain the transition from suicidal ideation to suicidal action and to derive screening points and intervention targets. Pediatric populations and non-suicidal self-injury (NSSI) are excluded.

The aims are: (i) to synthesize the available evidence on the FM–SB/suicide association; (ii) to examine the mediating and/or moderating role of neuropsychological correlates together with key clinical variables; and (iii) to identify and discuss methodological gaps (measurement heterogeneity, insufficient control of confounders, and low event rates) to inform longitudinal and mechanistic research priorities, as well as to guide screening and clinical practice.

## 2. Methods

### 2.1. Literature Search Strategy and Evidence Identification

Initially, the inclusion and exclusion criteria were defined, alongside the conceptual focus of the study (integration of the evidence on FM and SB with neuropsychological correlates and ideation-to-action frameworks). Subsequently, a targeted literature search was conducted in PubMed/MEDLINE, Web of Science (WOS), and Scopus. The last search was performed on 14 October 2025. Although the search and selection were structured, this work is a critical review and was not conducted as a PRISMA-guided systematic review; therefore, no preregistered protocol was used and we did not perform a formal risk-of-bias appraisal or a PRISMA flow diagram, or standardized study-by-study data extraction.

The search strategy was structured using Boolean operators (AND/OR) and combined controlled and free-text terms. In PubMed/MEDLINE, MeSH (Medical Subject Headings) descriptors were used when available, together with free-text terms; in Scopus and WOS, the syntax was adapted to the specificities of each platform. Terms were organized into three thematic blocks: (1) clinical condition (FM/chronic pain), (2) SB (ideation, planning, attempts, and suicide mortality), and (3) neuropsychological correlates and core constructs from ideation-to-action models, combined flexibly depending on the purpose of each search. Specifically, terms such as “fibromyalgia”, “chronic pain”, “suicide”, “suicidal ideation”, “suicide plan/suicide planning”, “suicide attempt”, “death by suicide/completed suicide”, and “suicide mortality” were considered, along with terms related to neuropsychological correlates and pain (e.g., “executive function”, “decision making”, “impulsivity”, “pain catastrophizing”, and “pain vigilance/attentional pain vigilance”) and core terms from ideation-to-action models (“interpersonal theory of suicide”, “integrated motivational–volitional model”, “three-step theory”, and “ideation-to-action”).

Two investigators (C.M.L.G. and S.M.) conducted independent searches. After that, titles and abstracts were reviewed for relevance, and those that did not meet the criteria and duplicates were excluded. Thereafter, the eligibility of the remaining articles was examined in detail. All full texts of the selected articles were retrieved and assessed according to the inclusion and exclusion criteria. In cases of disagreement, articles were re-evaluated and a final decision was reached by consensus; when disagreement persisted, C.M.L.G. made the final decision.

### 2.2. Eligibility Criteria

The following inclusion criteria were applied (institutional sources—e.g., National Institute of Statistics [INE] and World Health Organization [WHO]—were used solely for epidemiological contextualization and were not part of the eligibility process for included studies): (1) peer-reviewed empirical studies (cross-sectional and longitudinal designs); (2) theoretical reviews, systematic reviews and/or meta-analyses in adult populations with FM and/or chronic pain, as well as theoretical papers relevant to ideation-to-action models; (3) adult participants (≥18 years); (4) studies focused on suicidal ideation and/or suicidal action and their relationship with clinical and/or cognitive/neuropsychological variables relevant in FM and/or chronic pain, as well as key theoretical literature for ideation-to-action frameworks; (5) studies written in English or Spanish.

The exclusion criteria were: (1) pediatric participants (<18 years) to avoid developmental maturity-related biases in executive control and due to clinical differences in the presentation of pediatric FM; (2) studies focused exclusively on NSSI, given its phenomenological and motivational distinctiveness (absence of lethal intent) from SB; (3) commentaries, reports, letters, editorials, meeting/conference abstracts, and/or case reports; and (4) studies written in languages other than English or Spanish. This approach supports a critical synthesis of the available evidence without applying the exhaustiveness or formalized procedures of a PRISMA-guided systematic review.

## 3. Historical Background on the Study of Suicidal Behavior in Fibromyalgia

Suicide is a global public health problem whose complex and multifactorial nature demands coordinated responses from nations (Rizvi et al., 2017; WHO, 2025). Each year, approximately 727,000 people worldwide die by suicide (WHO, 2025). In Spain, the provisional 2024 data balance, published in June 2025 by the National Institute of Statistics (INE, 2025), places suicide as the second leading cause of external mortality, with 3846 deaths. Historically, the study of the suicide phenomenon in FM has taken a back seat to other chronic pain clinical conditions (e.g., trigeminal neuralgia [Adams et al., 2011; Fishbein et al., 2025], migraine [Giakas et al., 2023], temporomandibular pain [Han, 2018], and back pain [Ilgen et al., 2013]) despite the strong connection between persistent clinical pain and suicidal thoughts and behaviors (Rizvi et al., 2017). The current scientific literature suggests a consistent association between chronic pain and SB (Calati et al., 2015) and between FM and SB (Adawi et al., 2021; Treister-Goltzman & Peleg, 2023).

Given the broad conceptual nature of SB, this review encompasses both ideation (active/passive) with or without suicidal planning, as well as suicidal action—an umbrella term that, in this work, includes preparatory behaviors and suicide attempt (Crosby et al., 2011; Silverman et al., 2007)—while “completed suicide” is reserved for the fatal outcome. Non-suicidal self-injury (NSSI) is excluded due to the absence of lethal intent (Nock, 2010). Henceforth, SB is used as the general term that includes both ideation and suicidal action; regarding the latter, its components (preparatory behaviors and suicide attempt) will be separated when required by the study design (O’Carroll et al., 1996; Posner et al., 2011; Silverman et al., 2007).

Operationally, active ideation comprises thoughts of taking one’s life without a specific method or defined intent (non-specific active ideation), as well as thoughts with intent and/or suicidal planning (Posner et al., 2011, 2010). Passive ideation comprises thoughts related to the wish to be dead without including intent and/or suicidal planning—in this regard, suicidal planning refers to the formulation of the method, time, and/or location (Posner et al., 2010). Preparatory behaviors are defined as obtaining lethal means, rehearsing, or leaving messages and/or notifications without carrying out the act (Posner et al., 2010). A suicide attempt is self-injurious behavior that involves some degree of intent to die, regardless of lethality or outcome (it includes real, aborted, and interrupted attempts) (O’Carroll et al., 1996; Posner et al., 2010; Silverman et al., 2007). NSSI is defined as self-injurious behavior without the intent to die (Nock, 2010; Posner et al., 2010; Silverman et al., 2007).

This section aims to trace the evolution of SB and suicide in chronic pain, and specifically in FM, from the earliest works using indirect measurements up to contemporary risk models (e.g., variables such as hopelessness and/or perceived burden).

Thus, in the late 1990s, an initial review of the scientific literature indicated that chronic pain could constitute a risk factor for both SB and completed suicide (Fishbain, 1999). A subsequent review (Tang & Crane, 2006) confirmed and quantified this association: people with chronic pain had a two-to three-times higher risk of death by suicide, as well as higher prevalence of suicidal ideation and attempts, regardless of the pain subtype. Nevertheless, most of the included studies were cross-sectional, employing heterogeneous and often poorly standardized SB measures, and frequently failed to adequately control for the influence of depression or other confounding factors (e.g., insomnia). Additionally, for completed suicide, some prospective studies were developed (Macfarlane et al., 2001; Penttinen, 1995; Timonen et al., 2003), but they had methodological problems similar to the former: poor control of confounding variables, heterogeneous measures, and few recorded events, which underscored the need for more specific and consistent assessments and broader longitudinal studies.

In FM, the scientific literature on SB and completed suicide is less robust. Two pioneering studies on suicide in FM were conducted in a Danish and a US cohort, observing a higher risk of suicide in FM patients compared to the general and psychiatric populations (Dreyer et al., 2010; Wolfe et al., 2011). In Spain, Calandre and his team documented that 16.7% of patients with FM reported at least one previous suicide attempt (95% CI: 11.5–22.9), a proportion much higher than the estimated lifetime prevalence in the general population (2.7%) (Calandre et al., 2011). Subsequently, scientific evidence revealed that transdiagnostic factors (e.g., hopelessness, perceived burden) might explain the risk of SB more than the specific etiology of the pain (Lafuente-Castro et al., 2018). Furthermore, patients with FM have been reported to have up to a ten-fold higher risk of death by suicide than the general population in certain cohorts (McKernan et al., 2019). The first specific systematic review on the topic was published in 2020 (Levine & Horesh, 2020), which, along with a subsequent meta-analysis (Adawi et al., 2021), estimated high prevalence of suicidal ideation (approx. 30% [95% CI: 1.84–72.07]) and suicide attempts (approx. 6% [95% CI: 1.26–31.34]) and reported an odds ratio (OR) of 36.77 for “suicide risk” in FM patients versus controls (95% CI: 15.55–96.94), as well as a hazard ratio (HR) of 1.38 for suicidal events (95% CI: 1.17–1.71). The latest systematic review and meta-analysis of mortality in FM (Treister-Goltzman & Peleg, 2023) (to our knowledge) revealed suicide as a specific cause of death in this population with a standardized mortality ratio (SMR) of 3.37 (95% CI: 1.52–7.50), indicating that FM patients have more than three times the risk of dying by suicide compared to the general population. This study emphasizes the importance of suicidal ideation in this population and proposes its preventive integration into clinical practice. Collectively, this gap, coupled with clinical approaches that are excessively focused on mood—which may mask other relevant factors such as pain/interference, sleep, fatigue, cognition, and medication—highlights the need to update scientific evidence with standardized measures and contemporary risk models, with the goal of reducing the risk of suicide in FM patients.

This section provides an operational definition of SB adapted for FM and a critical overview of the evidence. However, persistent longitudinal and measurement gaps justify the adoption of ideation-to-action frameworks in the following sections.

## 4. Neuropsychological Correlates and Suicidal Behavior in Fibromyalgia: Integration with *Ideation-to-Action* Models (IPTS/3ST/IMV)

The ideation-to-action framework posits that suicide involves two distinct processes with partially different predictors: (a) the generation of suicidal ideation and (b) the progression from ideation to suicidal action (Klonsky et al., 2016, 2017, 2018; Klonsky & May, 2015).

Factors such as perceived hopelessness, depression, and most mental disorders act as predictors of suicidal ideation. Conversely, these factors do not accurately discriminate between those who only think about suicide (ideators) and those who attempt it (attempters). In contrast, factors like pain habituation, lower fear of death, and previous painful and/or provocative experiences (PPEs) act as key predictors in differentiating between ideators and attempters (Bayliss et al., 2022; Klonsky et al., 2016, 2017, 2018; Klonsky & May, 2015). In FM, this schema aligns with two differential profiles: motivational factors that sustain ideation (e.g., hopelessness, pain catastrophizing, attentional pain vigilance, fatigue, and non-restorative sleep), and volitional factors that facilitate the transition to action (e.g., impulsivity, deficits in inhibitory control, flexibility, and acquired capability due to painful/provocative experiences and exposure to healthcare procedures). This framework allows for the integration of FM’s neuropsychological correlates without confusing predictors of ideation with predictors of action (da Silva et al., 2024; Muñoz Ladrón de Guevara et al., 2018; Ordóñez-Carrasco et al., 2021).

Within this theoretical framework, the Interpersonal Theory of Suicide ([IPTS]; Joiner, 2005) postulates that the combination of thwarted belongingness and perceived burden, especially when accompanied by hopelessness, induces suicidal desire, while acquired capability facilitates the transition to suicidal action.

In FM, the coexistence of high attentional pain vigilance and high pain catastrophizing can amplify perceived burden (greater functional dependence) and thwarted belongingness (isolation and/or social stigma), which, in turn, fosters hopelessness and, consequently, suicidal desire. Furthermore, the combination of patient-reported cognitive difficulties like “fibro-fog” and findings of executive dysfunction (switching, planning) makes self-regulation of distress and the handling of daily demands difficult (simple, demanding, or changing tasks), thereby reinforcing this interpersonal circuit of the IPTS (Gálvez-Sánchez et al., 2018, 2020; Montoro & Reyes del Paso, 2015; Muñoz Ladrón de Guevara et al., 2018).

Perceived burden—the feeling of being a burden to others—shows robust associations with suicidal ideation and moderate associations with suicidal action (Batterham & Calear, 2021; Blais & Grimm, 2025). In FM, perceived burden can be increased by the daily functional dependence (Lempp et al., 2009), occupational limitations (Laroche et al., 2019), and caregiver burden (Ferahman et al., 2025). Additionally, recent scientific evidence suggests that gratitude may act as a protective factor and partially alleviate the clinical impact of FM (Carneiro et al., 2025a). Within ideation-to-action models, this positive resource may mitigate psychological pain and perceived burden, thereby attenuating the motivational pathway toward suicidal ideation. Thwarted belongingness—the feeling of isolation and/or social disconnection—is linked to suicidal ideation with a smaller magnitude than perceived burden and shows a secondary relationship with suicidal action (Blais & Grimm, 2025; Chu et al., 2017). In FM, thwarted belongingness is fueled by isolation and social stigma linked to chronic pain and the extensive medical-healthcare journey that patients typically navigate. In this context, hopelessness operates as a booster of suicidal desire (Chu et al., 2017). For FM patients, it is especially relevant due to the fluctuations in and unpredictability of the clinical course, possible perceived therapeutic failures, and clinical factors such as fatigue and sleep disturbances, among others.

Finally, acquired capability—defined as PPEs—would act as a differentiating process between ideators and attempters (Becker et al., 2020; Blais & Grimm, 2025; Chu et al., 2017). Impulsivity (Yilmaz & Tamam, 2018) and alterations in cognitive inhibition (Muñoz Ladrón de Guevara et al., 2018) in FM, combined with the experience of clinical factors like fatigue, clinical pain, and the continuous use of medication (e.g., opioid medication), could collectively act as facilitators of suicidal action. This pattern is consistent with cognitive profiles described in FM and with the distinction between ideation and action found in ideation-to-action models (Becker et al., 2020; Muñoz Ladrón de Guevara et al., 2018; Yilmaz & Tamam, 2018). However, small sample sizes, cross-sectional designs, and heterogeneous control of pain, sleep, and medication in cognitive tasks, among other clinical variables, persist, meaning the predictive validity from ideation to action remains limited in FM.

Another important theory within the ideation-to-action theoretical model is the Three-Step Theory ([3ST]; Klonsky & May, 2015). This theory hypothesizes, in its first step, that suicidal ideation results from the combined action of psychological pain and hopelessness. In the second step, social connectedness acts as a key protective factor against the progression of ideation; if that psychological pain and hopelessness outweigh social connectedness, ideation intensifies. In the third and final step, the escalation of ideation toward suicidal action is facilitated by dispositional factors (such as low fear of death and high pain tolerance), acquired factors (such as PPEs), and practical factors (such as knowledge, prior experience, and accessibility to lethal means). In addition to these classic distinguishing factors of the 3ST, various studies show dispositional factors like impulsivity and cognitive alterations in inhibitory control, cognitive flexibility, planning, decision-making, and general executive control—variables that the specialized suicide literature has linked to the progression from ideation to action (Burton et al., 2011; Richard-Devantoy et al., 2014; Saffer & Klonsky, 2018; Wang et al., 2017).

In its application to FM, the 3ST would operate as follows: in the first step, hopelessness (acting as a predictor of active ideation) and psychological pain (correlated with hopelessness and ideation) are consistently associated with ideation; patients with FM show high levels of both variables, presenting a greater vulnerability to ideation (da Silva et al., 2024; Ordóñez-Carrasco et al., 2021). In the second step, regarding the protective role of social connectedness, scientific evidence in FM reveals that weak informal support networks, and in particular, poorer marital adjustment are associated with increased ideation (Calandre et al., 2021; Lafuente-Castro et al., 2018). In the third and final step, the transition toward suicidal action in FM could be facilitated by predisposing dispositional and/or acquired factors such as increased impulsivity (Yilmaz & Tamam, 2018); poor inhibitory control; deficits in cognitive flexibility, decision-making, and planning (Muñoz Ladrón de Guevara et al., 2018); and, generally, poor executive control (Duschek et al., 2022). In sum, these neuropsychological correlates primarily operate in Step 3 of the 3ST; their impact on pain and hopelessness links them to Steps 1–2, and in the IMV/IPTS they correspond to volitional moderators/capability. These effects mainly come from small samples and cross-sectional designs, and should therefore be interpreted with caution.

The last theory within the ideation-to-action framework is the Integrated Motivational–Volitional Model ([IMV]; O’Connor, 2011; O’Connor & Kirtley, 2018). It comprises two relatively independent yet related phases. First, during the motivational phase, suicidal ideation develops. In this phase, various challenging life stressors (e.g., chronic pain, fatigue, sleep disturbances, psychiatric comorbidity) can generate feelings of defeat and/or humiliation, which, when combined with moderators such as inadequate coping strategies and problem-solving deficits, lead to feelings and thoughts of entrapment (e.g., the experience of “no way out” coupled with a strong urge to escape a state perceived as unbearable and exhausting). This entrapment, along with moderators such as low perceived belongingness, high perceived burden, pain catastrophizing, and negative future expectations, can lead ideators to view suicide as an effective and quick “escape” from these life challenges, which can crystallize into suicidal intention. Second, during the volitional phase, that intentionality can escalate to SB depending on the emergence of volitional moderators such as acquired capability, impulsivity, access to lethal means, and social imitation/learning; planning is considered in this work to represent high-severity ideation (not behavior), with proximal volitional relevance (O’Connor & Kirtley, 2018).

In the context of FM, it could be hypothesized that chronic pain, fatigue, sleep disturbances, psychiatric disorders, cognitive deficits and patient-reported cognitive difficulties (fibro-fog [e.g., see Bell et al., 2018; Samartin-Veiga et al., 2019; Wu et al., 2018]), and the usual healthcare and social stigmatization, increase defeat and humiliation. Under moderators such as pain-focused coping strategies, pain catastrophizing, and deficits in decision-making-based problem solving (e.g., in the Iowa Gambling Task [IGT; a task designed to assess decision-making under ambiguous or highly demanding situations] poorer learning—aberrant curves—and selection of less advantageous options are observed) (Gálvez-Sánchez et al., 2018, 2020; Montoro & Reyes del Paso, 2015; Muñoz Ladrón de Guevara et al., 2018), defeat can transform into entrapment (e.g., “no way out of the pain” coupled with the desire to escape that state perceived as unbearable and exhausting). In turn, low perceived belongingness, enhanced by social isolation resulting from chronic pain, and high perceived burden, enhanced by poorer marital adjustment, favor the crystallization of entrapment into suicidal intention. It is during the volitional phase that the transition from intention to SB occurs, facilitated by characteristic moderators in FM such as habituation to pain and constant exposure to medical procedures (acquired capability), in addition to impulsivity; sleep disturbances; executive control dysfunction; the domestic use of sedative, analgesic, and/or opioid medication (access to lethal means); and social imitation and/or learning, among others. This fit not only summarizes the IMV in FM but also generates operational predictions—that low belongingness/high burden and entrapment will explain ideation over and above pain, psychiatric comorbidity, pain catastrophizing, and negative expectations, while impulsivity, executive control dysfunction, acquired capability, and access to lethal means will explain the transition to SB—guiding longitudinal designs with mediation/moderation and focused clinical screening.

**Figure 1:**
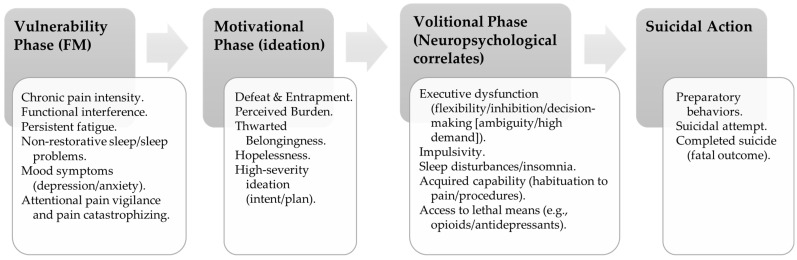
Integrated ideation-to-action model in fibromyalgia.

In summary, the IPTS, 3ST, and IMV models are integrated to differentiate predictors of ideation (hopelessness, perceived burden, thwarted belongingness) and action (impulsivity, acquired capability, access to means), identify priority screening variables, and define therapeutic targets; their transfer to FM is synthesized. Suicidal planning is conceptualized as high-severity ideation (not yet suicidal action), constituting a clear indicator of proximity to execution. Figure 1 summarizes hypothesized associations: FM vulnerability factors may contribute to motivational processes underlying suicidal ideation, including high-severity ideation with intent/a plan (planning is conceptualized as ideation). The transition from ideation/intention to suicidal action may be moderated by volitional factors (e.g., executive dysfunction, impulsivity, acquired capability, sleep disturbances/insomnia, and access to lethal means). Suicidal outcomes are operationalized as preparatory behaviors and suicide attempt; ‘completed suicide’ is reserved for the fatal outcome. The overarching methodological constraints are synthesized in Section 7.1.

## 5. Key Clinical Interactions: Mediation/Moderation Between Neuropsychological Deficits and Suicidal Behavior

This section aims to summarize, without duplication, how clinical variables frequently encountered in FM act as mediators and/or moderators between neuropsychological deficits and SB within the ideation-to-action framework (IPTS/3ST/IMV).

Pain and Functional Interference. These increase defeat/entrapment and intensify ideation (IMV–motivational phase); furthermore, greater interference is associated with medication use and impulsivity, which can facilitate the transition to action (IMV–volitional phase/IPTS–capability).

Sleep Disturbances (non-restorative sleep/insomnia). These potentiate ideation via fatigue and affective dysregulation (anxiety and/or depression) and are associated with poorer inhibitory control (additional impact on the volitional phase).

Fatigue. This increases the feeling of ineffectiveness and entrapment (ideation) and is linked to poorer executive control, favoring the transition to action in the presence of other volitional moderators.

Mood State/Psychiatric Comorbidity. These primarily predict ideation but do not adequately discriminate suicidal action; they should be considered as covariates to avoid overestimating associations.

Pain Catastrophizing and Attentional Pain Vigilance. These reinforce hopelessness and/or entrapment and rumination (ideation) and worsen decision-making under ambiguity or highly demanding situations.

Medication Use (including opioids in real-world practice). Although current clinical guidelines do not recommend opioids as a first-line therapy (Häuser et al., 2017; Macfarlane et al., 2017), first-line pharmacological options include duloxetine, milnacipran, pregabalin, and low-dose tricyclic antidepressants (e.g., amitriptyline) (Häuser et al., 2017). However, real-world evidence indicates a high prevalence of opioid use among FM patients (Wolfe et al., 2013; Muñoz Ladrón de Guevara et al., 2018, 2022). This discrepancy is clinically relevant, as opioid use is associated with cognitive side effects—such as sedation and executive dysfunction (Kurita et al., 2012)—and provides access and capability to lethal means (IMV/IPTS/3ST), increasing the risk of transition to SB.

Taken together, pain/interference, sleep, and fatigue amplify defeat/entrapment and, along with mood and pain catastrophizing/vigilance, align with predictors of ideation (motivational phase). Meanwhile, impulsivity and deficits in inhibitory control, together with the use of potentially lethal medications, align with acquired/practical capability and facilitate the transition to action (volitional phase). These axes should be modeled as mediators or moderators in models aligned with IPTS/3ST/IMV.

## 6. Assessment of Suicidal Behavior in Fibromyalgia: Instruments and Psychometric Evidence from Ideation to Completed Suicide

Traditionally, the assessment of SB in FM has been operationalized using standardized measures with heterogeneous psychometric properties, which hinders the comparison of results (Levine & Horesh, 2020). In this context, it is considered a priority to move toward more homogeneous assessment procedures in research and clinical practice by implementing the use of validated instruments with proven evidence of reliability, validity, and, whenever possible, sensitivity to change, which facilitate reproducibility, generalizability, and the comparison of results across studies. Below, the most commonly used instruments and their psychometric evidence are synthesized according to the definition of SB adopted in the present study; furthermore, specific methodological considerations for FM (e.g., possible symptom overlap between chronic pain and hopelessness) are discussed.

Columbia Suicide Severity Rating Scale ([C-SSRS]; Posner et al., 2011), Spanish adaptation by Al-Halabí et al. (2016). This scale consists of a semi-structured interview that assesses the severity, intensity, and frequency of ideation (active/passive), suicidal planning (high-severity ideation), preparatory behaviors and suicide attempt (suicidal action), and the lethality of the suicide attempt. Additionally, it records NSSI (Posner et al., 2011). Clinical administration is brief. It is also available in formats that cover assessment from recent periods (e.g., the last month) up to lifetime history. Regarding scoring and interpretation, ideation severity is quantified by the maximum level reached (on a five-point Likert scale of increasing severity—where 1 = wish to be dead and 5 = ideation with a specific plan and intent—). Ideation intensity is derived from a composite scale of five elements (frequency, duration, controllability, deterrents, and reasons for ideation), and its sum ranges from 2 to a maximum of 25 points when ideation is present. Suicidal action (preparatory behaviors and suicide attempt [including actual, aborted, or interrupted attempts]) is quantified by type and lethality, with no universal cutoff scores. Operationally, suicidal action elevates the risk above any level of ideation; if there is no suicidal action, ideation with suicidal intent and/or a plan (levels 4–5 [highest severity of ideation]) implies greater risk imminence (Posner et al., 2011). Overall, the Spanish version of this scale shows discrete-to-moderate psychometric properties: Cronbach’s α of 0.53 for the intensity subscale, moderate convergent and discriminant validity, and documented sensitivity to change, especially in intensity (Al-Halabí et al., 2016).

In FM, there are currently no specific validations of the C-SSRS. However, it is widely used in chronic pain (e.g., see [Legarreta et al., 2018; Skljarevski et al., 2010]) and chronic pain studies that consistently link pain catastrophizing to SB (e.g., see [Legarreta et al., 2018; Racine, 2018]). Given the high prevalence of pain catastrophizing in FM (Varallo et al., 2024), the use of the C-SSRS could be appropriate for the assessment of SB in FM, although its interpretation must be cautious due to the lack of a specific validation. Furthermore, in FM, as a consequence of the frequent comorbidity with depression and pain catastrophizing, ideation responses (e.g., “it’s unbearable, I don’t want to live like this”) may be overrepresented without necessarily implying suicidal action. Therefore, it is recommended to contrast the C-SSRS with measures of pain (Visual Analogue Scale [VAS]/Numeric Rating Scale [NRS]), mood (Beck Depression Inventory-II [BDI-II]/Patient Health Questionnaire-9 [PHQ-9]), and pain catastrophizing (Pain Catastrophizing Scale [PCS]), in addition to the clinical interview and temporal evolution.

Plutchik Suicide Risk Scale ([PSRS]; Plutchik et al., 1989), Spanish adaptation by Rubio et al. (1998). This is a brief instrument for suicidal risk screening that integrates indicators of ideation (current and lifetime), communication of intent, previous attempts, and affective/behavioral markers (depression/hopelessness, insomnia, loss of control/impulsivity, withdrawal, family history). Furthermore, it provides a profile of current risk by taking into account lifetime history (e.g., previous attempts, impulsivity, hopelessness), and not solely the intensity of ideation during a specific period (Plutchik et al., 1989). It is a self-administered scale consisting of 15 dichotomous items (Yes = 1 point/No = 0 points) with a total score ranging from 0 (no risk) to 15 points (maximum risk). The Spanish adaptation established a cut-off point of ≥6 to be considered “positive risk”. The reported psychometric properties of the Spanish validation show high internal consistency (Cronbach’s α = 0.90), high test–retest reliability of 0.89, and a high sensitivity of 74% and specificity of 95%, which allows for adequate discrimination between controls and patients. Using the same cut-off, the sensitivity and specificity are 88% for discriminating individuals who have a history of suicide attempt from those who do not (Rubio et al., 1998).

In FM, suicidal risk scores assessed using the PSRS were significantly associated with FM severity (evaluated via the Revised Fibromyalgia Impact Questionnaire [FIQR]) and with pain, anxiety, depression, and poor sleep quality; additionally, in this study, PSRS scores were higher for those who reported previous attempts (Calandre et al., 2011). Furthermore, a subsequent study compared FM patients with chronic-low-back-pain patients and pain-free controls, revealing a marked prevalence of ideation and very high suicide risk in FM patients (Jiménez-Rodríguez et al., 2014). In FM—and, by extension, in chronic pain—the PSRS functions as an agile and pragmatic screening tool due to its brevity and ease of scoring. However, it does not discriminate between subtypes of behavior without the detail of clinical interviews (e.g., real vs. interrupted/aborted attempts), and some items may overlap with hopelessness/depression—frequent in FM—potentially raising false positives if interpreted out of context. Therefore, it is recommended to systematically triangulate with a structured clinical interview (e.g., C-SSRS) to characterize ideation, planning, preparatory behaviors, and attempt; supplement it with measures of mood (BDI-II/PHQ-9), pain (VAS/NRS), sleep, and pain catastrophizing; and explicitly document the recall period, existence of planning, and deterrents, especially during phases of intense pain (Al-Halabí et al., 2016). Overall, the available evidence positions the PSRS as a reliable and rapidly applied screening tool in the Spanish general population and, in particular, useful for stratifying risk in FM (correlation with FIQR and a higher rate of “positive risk” compared to controls), with results replicated in cohorts and comparative studies (Calandre et al., 2011; Jiménez-Rodríguez et al., 2014). Nonetheless, its limitations are those common to suicidal risk screening instruments—possible overlap with depressive/anxious symptoms and still limited evidence on longitudinal sensitivity to change—so its use must always be integrated within a structured clinical assessment and safety protocols (Rubio et al., 1998).

Item 9 of the Beck Depression Inventory-II ([BDI-II]; Beck et al., 1996), Spanish adaptation by Sanz et al. (2003). Traditionally, within the context of SB, Item 9 of the BDI-II has been widely used as a measure of ideation (mainly focused on active ideation according to the gradation in the original scoring, where 0 = “I don’t have thoughts of killing myself” and 3 = “I would kill myself if I had the chance”). It constitutes a very brief indicator extracted from a broader 21-item self-report measure (Beck et al., 1996), which can be used as a spot marker (1 item). Although it is not validated as an independent instrument to assess risk, its administration takes less than one minute if used in isolation, with the complete inventory needing five to ten minutes to be accomplished. In terms of its time frame, it refers to the last two weeks (including today) and is scored on a Likert-type scale of 0–3 points of increasing severity (where 0 = absence of ideation and 3 = intense ideation with intention/purpose). The BDI-II lacks universal cut-off points; however, operatively, any score of ≥ 1 indicates the presence of ideation, and scores of 2–3 suggest greater clinical imminence, meaning any score of ≥ 1 requires immediate clinical assessment. Since it is a single item, estimating Cronbach’s α is not applicable. Evidence supports the construct validity of the BDI-II and its extended use for monitoring, and Item 9 has been used as an indicator of ideation. Convergence with other ideation measures (e.g., C-SSRS and Item 9 of the PHQ-9 [Kroenke et al., 2001; Posner et al., 2011]) is conceptually consistent, although specific item-by-item evidence is limited. The Spanish version shows good reliability and validity for depression, having not been designed to predict suicidal behavior on its own. Furthermore, the BDI-II shows sensitivity to change at the scale level; the sensitivity of Item 9 in isolation must be interpreted with caution.

In FM, its use is frequent and consistently associated with greater clinical burden—more depression, pain catastrophizing, pain/disability, and poorer quality of life—with high prevalence of ideation and robust relationships with psychological variables (depression, anxiety, sleep, pain catastrophizing) documented in FM samples (Calandre et al., 2015; Ordóñez-Carrasco et al., 2021; Varallo et al., 2024). As an additional advantage to those mentioned above, its minimal respondent burden and wide availability in studies that already administer the BDI-II are notable (e.g., see [Calandre et al., 2015; Ordóñez-Carrasco et al., 2021]). Its limitations include overlap with depression and chronic pain distress—with possible overrepresentation of verbalizations like “I don’t want to go on like this” without suicidal action—the absence of information on planning or behaviors, and the lack of universally accepted cut-off scores, meaning it does not replace a structured risk interview. In practice, it is recommended to contrast it with measures of pain (VAS/NRS), mood (BDI-II/PHQ-9), and pain catastrophizing (PCS), in addition to the clinical interview and temporal evolution.

Suicide Behaviors Questionnaire—Revised ([SBQ-R]; Osman et al., 2001), Spanish adaptation by Gómez-Romero et al. (2021). This consists of a multicomponent self-report measure of SB (lifetime history, recent ideation, threat of attempt, and future probability), which is quick and self-administered (estimated time between one and two minutes). It quantifies the frequency of ideation from the “*lifetime*” up to the “*last 12 months*”. The total score ranges from 3 to 18, with no universal cut-off scores, although cut-off points of ≥7 in the general population and ≥ 8 in clinical settings are typically used, according to the original validation. Overall, the Spanish version shows discrete-to-moderate psychometric properties: acceptable internal reliability (Cronbach’s α = 0.80 in university students), high short-term temporal stability (test–retest = 0.88), and moderate-magnitude convergent and criterion validity. Sensitivity to change is plausible due to its brief format, but must be interpreted with caution because longitudinal studies in Spanish are still limited (Gómez-Romero et al., 2021).

In FM, the scientific literature documents ideation profiles linked to depression/anxiety, poorer sleep quality, and greater functional impact (Triñanes et al., 2015), and recent studies confirm that these profiles have a high prevalence and overlap with psychiatric comorbidity, disability, and sleep problems (Levine & Horesh, 2020). More broadly, in chronic pain, the association with SB is consistent in reviews and large cohorts (Ilgen et al., 2013; Tang & Crane, 2006). The risk appears to increase when pain is moderate-to-severe and, additionally, when insomnia coexists (Ashrafioun et al., 2019, 2021). A recent meta-analysis on chronic pain estimates high proportions of ideation within short time frames (Kwon & Lee, 2023). In this context, the SBQ-R offers practical advantages (speed, multimodal coverage, and wide availability) with minimal respondent burden. Among its limitations, it may overlap with depressive symptoms reactive to pain; furthermore, it does not discriminate planning or specific behavioral modalities in detail, and it does not replace a structured risk interview. In clinical and research practice in FM, its interpretation should be triangulated with pain intensity (VAS/NRS), mood state (BDI-II/PHQ-9), sleep, and pain catastrophizing, along with the clinical interview and temporal evolution. This is especially relevant when affective comorbidity exists, which can amplify the overall risk score (Calandre et al., 2011; Levine & Horesh, 2020; Triñanes et al., 2015).

Finally, in FM, completed suicide is documented through mortality registries and population cohorts. This is achieved by linking the cohort to the official death registry and identifying deaths whose underlying cause on the certificate is coded in the International Classification of Diseases—Tenth Edition [ICD-10] as intentional self-harm (X60–X84) or resulting from the sequelae of self-harm (Y87.0) (WHO, 2016). There are no items or direct clinical applications, and the assessment period is determined by longitudinal follow-up. It is not a scale; there are no scores or cut-off points: the outcome is expressed in rates and comparative measures—SMR (standardized mortality ratio: observed/expected after standardization), HR (hazard ratio: ratio of instantaneous risks over time, typically via Cox model), and RR (risk ratio: cumulative risk between groups), where values > 1 reflect an excess compared to the reference population.

In FM, these studies show excess mortality due to suicide with similar overall total mortality; reviews confirm the increased risk and methodological heterogeneity (Adawi et al., 2021; Dreyer et al., 2010; Levine & Horesh, 2020; Treister-Goltzman & Peleg, 2023; Wolfe et al., 2011).

Table 1 provides a summary of the instruments’ main features, properties, and constraints. A consolidated critical appraisal of these transversal strengths and limitations is provided in Section 7.1.

## 7. Methodological Challenges and Clinical Implications

### 7.1. Critical Appraisal of the Evidence Base

As this work is a critical review, it is essential to state that these clinical implications must be read with caution due to the non-systematic nature of the review.

The evidence base in FM relies largely on small samples and cross-sectional designs, particularly in the neuropsychological field. This body of work is informative for identifying correlates; however, it does not allow temporal inference or robust predictive validity to discriminate the transition from ideation (motivational component) to suicidal action (volitional component). The application in FM remains limited by a constrained longitudinal evidence base, heterogeneous operational definitions of ideation/action, low event numbers, and the absence of specific validations. Prospective studies are required with standardized criteria and direct contrasts of these hypotheses, along with trials that assess whether intervention on the identified targets reduces the transition from ideation to action.

A further limitation concerns heterogeneity in the operationalization of SB components (ideation, suicidal planning, preparatory behaviors, and suicide attempt) and in the reference periods used across instruments, which hampers comparability across studies. In parallel, approaches that focus predominantly on mood may mask other clinically relevant factors in FM (pain intensity and interference, sleep, fatigue, cognition, and medication), underscoring the need to interpret associations with adequate consideration of covariates.

From an assessment perspective, several instruments are useful for screening/monitoring; nevertheless, in FM it is essential to account for overlap between depression/pain distress and core FM symptoms. In this context, ideation responses may be overrepresented without necessarily implying suicidal action, and some items overlap with hopelessness/depression, potentially raising false positives if interpreted out of context; additionally, affective comorbidity can amplify the overall risk score. Therefore, in both clinical and research practice, findings should be systematically triangulated with pain intensity (VAS/NRS) and interference, mood state (BDI-II/PHQ-9), sleep, and pain catastrophizing/attentional pain vigilance, together with the clinical interview and temporal evolution.

In summary, in FM, instruments with Spanish versions and extended use are currently available (C-SSRS, PSRS, BDI-II Item 9, SBQ-R), with acceptable psychometric evidence for screening/monitoring and replicated associations with clinical burden (depression/anxiety, pain catastrophizing, pain/sleep). Furthermore, registries/cohorts have documented excess mortality due to suicide compared to the reference population. However, specific validations in FM (C-SSRS/SBQ-R/PSRS) with robust sensitivity to change and predictive validity for attempts or death are still lacking.

### 7.2. Clinical Implications and Future Perspectives

As a central clinical contribution of this critical review, for the first time, the *ideation-to-action* theoretical models (IPTS/3ST/IMV) of SB are integrated with clinical and neuropsychological correlates in FM, which allows for a clear differentiation between ideators (those who think about it) and attempters (those who act).

Thus, ideation—the motivational component of SB—can be primarily explained by hopelessness, perceived burden, and thwarted belongingness, intensified by pain, fatigue, non-restorative sleep, catastrophizing, and attentional pain vigilance. On the other hand, the transition to action—the volitional component of SB—can be explained by impulsivity, deficits in executive control (inhibition, flexibility, and decision-making under ambiguity or highly demanding situations), acquired capability due to repeated exposure to pain and medical procedures, and access to lethal means (e.g., domestic access to opioid medication).

Operationally, and to explicitly mirror this motivational versus volitional logic, this integration is clinically articulated across three main axes in FM.

Axis 1. Phase delineation (motivational ideation versus volitional action).

The first axis consists of clearly separating the ideation phase (the motivational component of SB = the desire to die) from the suicidal action phase (the volitional component of SB = capability/self-lethal execution). In FM it is essential to account for overlap between depression/pain distress and core FM symptoms. In this context, ideation responses may be overrepresented without necessarily implying suicidal action, and some items overlap with hopelessness/depression, potentially raising false positives if interpreted out of context; additionally, affective comorbidity can amplify the overall risk score.

Axis 2. Phase-aligned screening and profiling (motivational versus volitional profiles).

The second axis consists of establishing a differentiating clinical screening process that allows for the evaluation of overall SB risk. This will be done through the use of measurement instruments such as C-SSRS, PSRS, SBQ-R, and Item 9 (BDI-II), systematically contrasting their results with pain and attentional pain vigilance (interference), mood state, and pain catastrophizing. This contrasting is done, in turn, to discriminate motivational profiles from volitional profiles, thereby guiding clinical decision-making. Accordingly, when ideation is detected, the screening should be complemented by an explicit assessment of *ideation-to-action* transition indicators, including suicidal planning with intent (conceptualized as high-severity ideation), preparatory behaviors, suicide attempt, and access to lethal means (e.g., domestic access to opioid medication), together with the volitional correlates highlighted in this review (impulsivity and deficits in executive control).

Axis 3. Phase-tailored intervention (targeting the predominant SB component).

The third axis consists of directing intervention toward the predominant SB component: if the motivational component dominates, the intervention should target hopelessness, perceived burden, and isolation/social connectedness through personalized pain management (e.g., Acceptance and Commitment Therapy [see (Eastwood & Godfrey, 2024]); social reconnection via the involvement of informal social networks, partners, and/or family; and psychoeducation for sleep disturbances, fatigue, and pain catastrophizing and attentional vigilance (reframing and attentional redirection), in addition to problem-solving training. Conversely, if the volitional component dominates, the intervention target shifts to the application of a safety plan; restriction of access to lethal means (e.g., control and/or withdrawal of opioid medication); environmental structuring (e.g., restructuring and establishing daily routines and behaviors regarding the FM patient’s environment); mitigation of suicidal action implementation intention (e.g., training in warning signs: “If I observe myself looking for opioid medication, then I will inform my partner/family member/someone close and seek urgent help”); and neuropsychological rehabilitation programs that increase the executive capability of cognitive flexibility and inhibition, behavior planning, and advantageous decision-making in highly demanding and/or ambiguous situations.

With regard to future perspectives, longitudinal cohorts with sufficient sample size are required that allow for the analysis of the risk of suicidal action throughout follow-up and that, furthermore, explicitly differentiate ideation (motivational) from action (volitional). It is likewise necessary to standardize the operationalization of the different components of SB—that is, ideation, suicidal planning, preparatory behaviors, and suicide attempt—and to use comparable reference periods between assessment instruments, in order to improve comparability across studies and the interpretation of results. In turn, it would be advisable to establish a standardized neuropsychological battery that includes executive control variables such as inhibitory and cognitive flexibility, planning, and decision-making under ambiguous or highly demanding situations, and to systematically incorporate mediation and moderation analyses aligned with the *ideation-to-action* models (IPTS/3ST/IMV). It is also highly necessary to validate the most frequently used tools for SB assessment in FM (C-SSRS, PSRS, and SBQ-R). Finally, randomized clinical trials are needed to determine whether intervening with the motivational component of SB (hopelessness, thwarted belongingness, and perceived burden) and the volitional component (impulsivity, deficits in executive control, acquired capability due to repeated exposure to pain and medical procedures, and access to lethal means) buffers the transition from ideation to suicidal action, through interventions focused on pain reduction in FM.

Finally, given the multifactorial nature of SB in FM, prevention is unlikely to be effective if clinical management is delivered in parallel rather than integrated. Operationally, the implementation of the three main axes outlined above requires coordination between Rheumatology and Pain Medicine—aimed at optimizing symptom control and function (pain/interference, sleep, fatigue) and systematically reviewing medication exposure and access to lethal means (e.g., domestic access to opioid medication); Psychiatry, to treat psychiatric comorbidity that primarily fuels the motivational component of SB and can amplify the overall risk score; and Psychology, to intervene with the mechanisms highlighted by ideation-to-action models by targeting cognitive–emotional drivers (e.g., perceived burden, thwarted belongingness, catastrophizing) and supporting the rehabilitation of executive control to reduce the likelihood of transition from ideation to action. In this context, focused clinical screening, together with the clinical interview and temporal evolution, is essential to guide phase-tailored intervention and timely referral when needed.

## 8. Conclusions

This critical review integrates, for the first time, the *ideation-to-action* models (IPTS/3ST/IMV) with clinical and neuropsychological correlates that may allow for discrimination between ideation (motivational component) and suicidal action (volitional component) when assessing SB in FM. Distinguishing between the ideation component (hopelessness, perceived burden, thwarted belongingness, entrapment, pain, attentional vigilance and pain catastrophizing, sleep, and fatigue) and the action component (impulsivity, deficits in executive control, acquired capability due to repeated exposure to pain and medical procedures, and access to lethal means) might be essential, as this separation may facilitate the operationalization of the clinical approach. Its operationalization might be structured around three axes: (1) explicitly separating ideation (motivational) from suicidal action (volitional); (2) establishing a differentiating clinical screening with C-SSRS, PSRS, SBQ-R, and BDI-II Item 9, using comparable reference periods and contrasting with pain/interference, mood state, and pain catastrophizing/attentional vigilance; and (3) tailoring the intervention to the identified profile: if ideation (motivational) predominates—hopelessness and the burden–thwarted belongingness binomial—focus might be placed on reconnection and psychoeducation, and on optimizing pain management, sleep, and fatigue management; if action (volitional) predominates—plan/means/impulsivity/executive control—priority might be given to a safety plan, means restriction, medication review, environmental structuring, and the rehabilitation of executive control.

## Figures and Tables

**Table 1:** Summary of suicide behavior assessment instruments in fibromyalgia.

Instrument	Assessed Factors	Scoring	Psychometric Properties	Use in FM
C-SSRS (Posner et al., 2011)	Ideation severity/intensity (passive/active), high-severity ideation (intent/plan), preparatory behaviors, suicide attempt (actual/aborted/interrupted) and lethality; includes an NSSI module.	Ideation severity: 1–5-point Likert scale. Intensity: sum of 5 elements (range 2–25). Ideation intensity sum range of 2–25 when ideation is present. Suicidal action type and lethality; no universal cut-offs; any action elevates risk above ideation, and ideation levels 4–5 imply greater imminence in the absence of action.	Spanish version: discrete-to-moderate psychometrics; alpha = 0.53 (intensity subscale); moderate convergent and discriminant validity; documented sensitivity to change (especially intensity).	No FM-specific validation; widely used in chronic pain. Useful to distinguish ideation (including intent/plan) from preparatory behaviors/attempts. Interpret cautiously due to symptom overlap (pain distress, depression, catastrophizing) and triangulate with pain (VAS/NRS), mood (BDI-II/PHQ-9), sleep and catastrophizing (PCS) measures, plus clinical interview and temporal evolution.
PSRS (Plutchik et al., 1989)	Integrating ideation (current and lifetime), communication of intent, previous attempts, affective/behavioral markers (depression/hopelessness, insomnia, loss of control/impulsivity, withdrawal), and family history; profiles current risk incorporating lifetime history.	15 dichotomous items. Range: 0–15. Cut-off point: ≥ 6 = positive risk.	Cronbach’s α: 0.90. Test–retest: 0.89. Sensitivity 74%/Specificity 95%. Using the same cut-off, sensitivity/specificity 88% for discriminating history of attempts.	Associated with FIQR severity, pain, anxiety/depression and poor sleep quality; higher in patients reporting previous attempts. Efficient screening, but limited discrimination of behavioral subtypes and potential symptom overlap; complement with a structured interview (e.g., C-SSRS).
BDI-II (Item 9) (Beck et al., 1996)	Single-item marker of active suicidal ideation extracted from a depression inventory (spot indicator).	0–3 over last 2 weeks (including today). Any score ≥ 1 warrants immediate clinical assessment; 2–3 suggests greater imminence.	Single item (internal consistency not applicable). BDI-II has validity/reliability for depression; Item 9 not validated as standalone risk tool. Item-level convergence is conceptually consistent, but evidence is limited; interpret change cautiously.	Frequently used and associated with greater clinical burden (depression, pain catastrophizing, pain/disability, poorer quality of life). Limitations: overlap with pain-related distress; provides no information on planning/behaviors and lacks universal cut-offs; does not replace a structured risk interview; triangulate with pain, mood, catastrophizing measures and temporal evolution.
SBQ-R (Osman et al., 2001)	Very brief self-report: lifetime attempts, recent ideation, threat of attempt, future probability. Captures time windows from lifetime to last 12 months.	4 items (range 3–18). No universal cut-offs; typically: ≥7 (general population) and ≥8 (clinical settings) in original version.	Cronbach’s α: 0.80. Test–retest: 0.88 (Spanish version). Moderate convergent/criterion validity. Sensitivity to change plausible; interpret cautiously (limited longitudinal Spanish evidence).	Linked to depression/anxiety, poor sleep quality and functional impact in FM/chronic pain cohorts; minimal respondent burden. Limitations: overlap with depressive symptoms reactive to pain; limited detail on planning/behavioral modalities; triangulate with structured interview and pain/mood/sleep/catastrophizing measures.
Mortality Registries (ICD-10)	Completed suicide (fatal outcome) in administrative registries/cohort linkage.	Codes: intentional self-harm X60–X84; sequelae Y87.0. Reported as rates and comparative measures (SMR/HR/RR).	Not applicable (outcome data source; not a psychometric scale).	Clinical suitability: Not for screening (epidemiological outcome). Population-level excess mortality in FM; complements individual-level instruments.

Note I. BDI-II: Beck Depression Inventory-II; C-SSRS: Columbia Suicide Severity Rating Scale; FM: fibromyalgia; ICD-10: International Classification of Diseases—Tenth Edition; NSSI: non-suicidal self-injury; PHQ-9: Patient Health Questionnaire-9; PSRS: Plutchik Suicide Risk Scale; SBQ-R: Suicide Behaviors Questionnaire—Revised. Note II. Psychometric properties refer exclusively to validation studies of the instruments in Spanish (when available). BDI-II: Beck Depression Inventory-II; C-SSRS: Columbia Suicide Severity Rating Scale; FIQR: Revised Fibromyalgia Impact Questionnaire; FM: fibromyalgia; ICD-10: International Classification of Diseases—10th Edition; NSSI: non-suicidal self-injury; PCS: Pain Catastrophizing Scale; PHQ-9: Patient Health Questionnaire-9; PSRS: Plutchik Suicide Risk Scale; SBQ-R: Suicide Behaviors Questionnaire—Revised; SMR: standardized mortality ratio; HR: hazard ratio; RR: relative risk; VAS: Visual Analogue Scale; NRS: Numeric Rating Scale.

## Data Availability

No new data were created or analyzed in this study. All data supporting the findings of this study are available within the published articles cited in the references.

## Abbreviations

The following abbreviations are used in this manuscript:

FM	Fibromyalgia
SB	Suicidal Behavior
IPTS	Interpersonal Theory of Suicide
3ST	Three-Step Theory
IMV	Integrated Motivational–Volitional Model
C-SSRS	Columbia Suicide Severity Rating Scale
PSRS	Plutchik Suicide Risk Scale
BDI-II	Beck Depression Inventory-II
SBQ-R	Suicide Behaviors Questionnaire—Revised
NSSI	Non-Suicidal Self-Injury
WHO	World Health Organization
INE	Instituto Nacional de Estadística
SMR	Standardized Mortality Ratio
PPEs	Previous Painful and/or Provocative Experiences
IGT	Iowa Gambling Task
VAS	Visual Analogue Scale
NRS	Numeric Rating Scale
PHQ-9	Patient Health Questionnaire-9
PCS	Pain Catastrophizing Scale
FIQR	Revised Fibromyalgia Impact Questionnaire
ICD-10	International Classification of Diseases—Tenth Edition
HR	Hazard Ratio
RR	Risk Ratio
OR	Odds Ratio
